# Magnetically Induced Switching of Circularly Polarized Luminescence Using Electromagnets

**DOI:** 10.3390/molecules30112426

**Published:** 2025-05-31

**Authors:** Yoshitane Imai, Kota Fukuchi, Yoshihiko Yanagihashi, Satoko Suzuki

**Affiliations:** 1 Department of Applied Chemistry, Faculty of Science and Engineering, Kindai University, 3-4-1 Kowakae, Higashi-Osaka 577-8502, Osaka, Japan; 2 JASCO Corporation, 2967-5 Ishikawa-machi, Hachioji 192-8537, Tokyo, Japan

**Keywords:** circularly polarized luminescence, electromagnet, perovskite quantum dots, switching

## Abstract

Intense circularly polarized luminescence is crucial for high-performance electroluminescent, optoelectronic, and photonic devices. This study investigates the magneto-chiral characteristics of two achiral soluble diamagnetic perovskite-type PbQDs. Magnetic fields of 158 and 198 mT are applied using an electromagnet in a toluene solution at 25 °C. Both PbQDs show a magnetic circularly polarized luminescence magnitude of approximately 10^−3^ within the (480 to 580) nm wavelength range. The strength of the magnetic circularly polarized luminescence increases with the intensity of the applied magnetic field. Furthermore, the study demonstrates rapid and reversible switching of the rotation direction of the magnetic circularly polarized luminescence when the magnetic poles are rapidly changed. These results suggest that the direction (right- and left-rotating light) and circular polarization of circularly polarized luminescence (CPL) from circularly polarized perovskites can be alternately and freely controlled by applying an external magnetic field with an appropriate direction and strength.

## 1. Introduction

Recently, strong circularly polarized luminescence (CPL) from organic, organic–inorganic, and inorganic luminophores has become an essential element of advanced functional dyes, high-performance electroluminescent materials, and photonic device materials. However, materials that emit strong CPL from the aforementioned luminophores require a high quantum yield (Φ_F_) and anisotropy factor (g_CPL_) due to the magnitude and orientation of their magnetic and electric dipole moments.

CPL can be categorized into right- and left-rotating CPL, generally requiring two types of enantiomeric optically active CPL materials. By altering the external environment, such as the solvent and temperature, around an optically active CPL material, both right- and left-rotating CPL can be emitted from the same optically active CPL material; however, the versatility of this method is considerably low. Additionally, this approach makes it challenging to instantaneously switch the rotation direction of CPL.

In optically active materials capable of generating CPL, organic CPL materials benefit from the diversity of molecular design through organic synthesis [1,2,3,4,5,6,7,8,9,10,11,12,13], whereas inorganic CPL materials have the advantages of easily controlling the optical properties by modifying constituent atoms or particle sizes, along with high thermal stability and durability [14,15]. However, as mentioned above, changing the external environments of both organic and inorganic CPL materials makes it difficult to instantaneously switch the rotation direction of CPL.

Moreover, CPL cannot be extracted from optically inactive organic, organic–inorganic, and inorganic luminophores through light excitation. However, by applying an external magnetic field using permanent magnets in a Faraday configuration and then performing light excitation, CPL can be extracted from these optically inactive luminophores. This phenomenon is called magnetically induced CPL (MCPL), which can be considered an excited version of magnetic circular dichroism [16,17,18,19,20,21,22,23,24]. Additionally, the direction of the MCPL can be controlled by reversing the direction of the applied magnetic field, thereby effectively swapping the north and south poles, and is an added advantage of the magnetic field-induced CPL.

Recently, we successfully extracted CPL from two types of optically inactive perovskite quantum dots (PVQDs), CH_5_N_2_PbBr_3_ (hereinafter referred to as PVQD-1) and CsPbBr_3_ (hereinafter referred to as PVQD-2), by applying an external 1.7 T magnetic field using permanent magnets in a Faraday configuration and conducting light excitation, both in dilute solutions and solid-state [25,26]. However, this permanent magnet did not enable instantaneous and reversible switching of the CPL rotation direction of these optically inactive PVQDs.

In this study, we aimed to instantaneously switch the direction of the magnetic field, that is, the rapid inversion of the N and S poles, by continuously changing the direction of the current flowing through the electromagnet. This approach targeted the instantaneous switching of the CPL rotation direction, i.e., the sign of the MCPL spectra. Additionally, we attempted to extract high-rotation MCPL from PVQD-1 and PVQD-2 by applying weak external magnetic fields of 158 and 198 mT using the electromagnet. The capability of CPL material for instantaneously switching the CPL rotation direction is considered essential for the future development of functional switching CPL materials.

## 2. Results and Discussion

**Figure 1:**
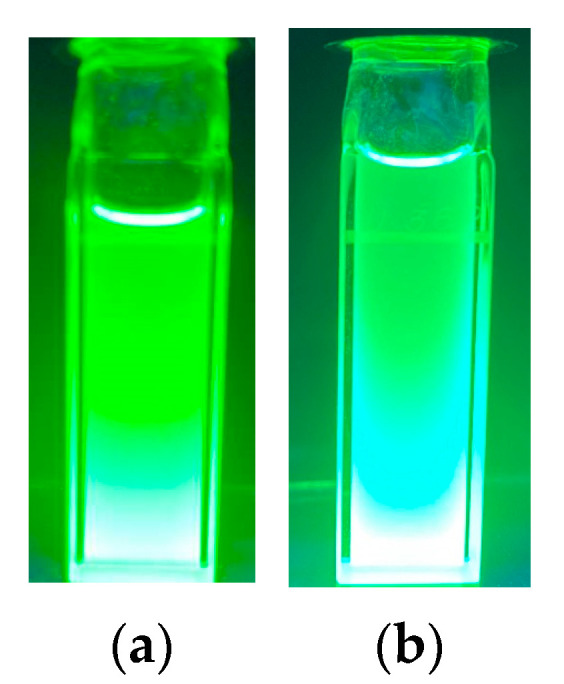
Photographs showing the photoluminescence emitted from (**a**) CH_5_N_2_PbBr_3_ quantum dots (PVQD-1) and (**b**) CsPbBr_3_ quantum dots (PVQD-2) in a 1.0 × 10^−4^ M toluene solution when the samples are excited with light (365 nm) from below.

As aforementioned, magnetically induced chiroptical properties of two types of optically inactive PVQDs, namely PVQD-1 and PVQD-2, were examined (see Figure 1). PVQD-1 was composed of formamidinium (CH_5_N_2_^+^) ions, whereas PVQD-2 consisted of cesium (Cs^+^) ions, both without chiral organic ligands. Despite having highly similar core sizes (approximately 10 nm), the surrounding constituent atoms around Pb differed. Figure 1 shows the photoluminescence emitted from PVQD-1 and PVQD-2 in 1.0 × 10^−4^ M toluene at an excitation wavelength of 365 nm at 25 °C.

**Figure 2:**
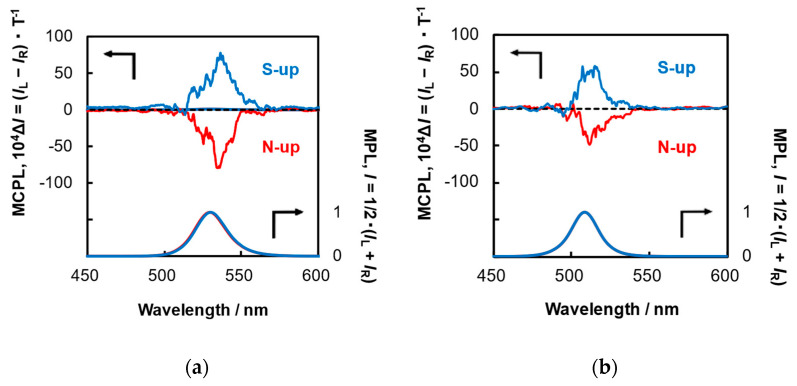
Magnetically induced circularly polarized luminescence (MCPL) (upper spectrum in each panel) and magnetic photoluminescence (MPL) (lower spectrum in each panel) spectra of (**a**) PVQD-1 (λ_ex_ = 398 nm) and (**b**) PVQD-2 (λ_ex_ = 360 nm), in dilute toluene (1.0 × 10^−3^ M) under N-up (red lines) and S-up (blue lines) Faraday configurations within a magnetic field of 158 mT at 25 °C.

To achieve reversible, continuous CPL rotation direction switching, i.e., reversible continuous magnetic field direction switching, an electromagnet with a Helmholtz coil was used as the magnet instead of a permanent magnet. For continuous switching of the magnetic field direction, the direction of the current flowing in the Helmholtz coil must be changed reversibly and continuously. The maximum value of the generated magnetic field was 158 mT to prevent the generation of a reverse current. Therefore, the MCPL characteristics at an applied magnetic field of 158 mT were investigated. Even when PVQD-1 was photoexcited in a weak magnetic field of 158 mT at 25 °C, the quantum dots effectively emitted strong MCPL. When the direction of the applied magnetic field [N-up (N → S) or S-up (S → N)] was altered, PVQD-1 displayed nearly mirror-image MCPL spectra at wavelengths corresponding to magnetic photoluminescence (MPL) (upper panel, as shown in Figure 2a).

In the presence of an external magnetic field of 158 mT, the sign of the MCPL spectrum is solely determined by the N-up and S-up directions in a Faraday configuration; negative (−) MCPL (red line) was observed in the N-up configuration, whereas positive (+) MCPL (blue line) was recorded in the S-up configuration. Table 1 provides the characteristics of MCPL for PVQD-1 in dilute toluene (1.0 × 10^−3^ M) at 158 mT. The MCPL wavelengths (λ_MCPL_) were nearly identical in the N-up and S-up configurations, measuring 535 nm for N-up and 537 nm for S-up configurations (refer to Table 1). These MCPL originate from the band gap between the Pb exciton of PVQD-1 and the surrounding constituent atoms such as Br, with the MCPL efficiency of PVQD-1 values of the order of 10^−3^, as listed in Table 1.

To further explore the influence of cations on the properties of MCPL when subjected to an external magnetic field of 158 mT, we examined the MCPL and MPL properties of PVQD-2 quantum dots in a toluene solution. The findings indicate that PVQD-2 exhibits strong MCPL even under a magnetic field of 158 mT, comparable to its performance under a stronger 1.7 T magnetic field, as illustrated in Figure 2b. The MPL spectra for PVQD-2 in the N-up and S-up orientations were similar, as observed with PVQD-1, with its MCPL wavelength measured at 512 nm for N-up and 516 nm for S-up configurations (see Table 1). Under the 158-mT magnetic field, nearly mirror-symmetric MCPL spectra were recorded along both the N-up and S-up Faraday configuration, and the signs were consistent with those observed for PVQD-1. As evident from Table 1, the MCPL efficiency of PVQD-2 was of the same order as that of PVQD-1, i.e., approximately 10^−3^ (see Table 1).

These findings indicate that CPL can be generated from achiral PVQD luminophores at 25 °C, even in an external magnetic field of 158 mT. Additionally, the direction of rotation of the MCPL in PVQD luminophores can be fully manipulated, even with a weak external magnetic field of 158 mT.

**Figure 3:**
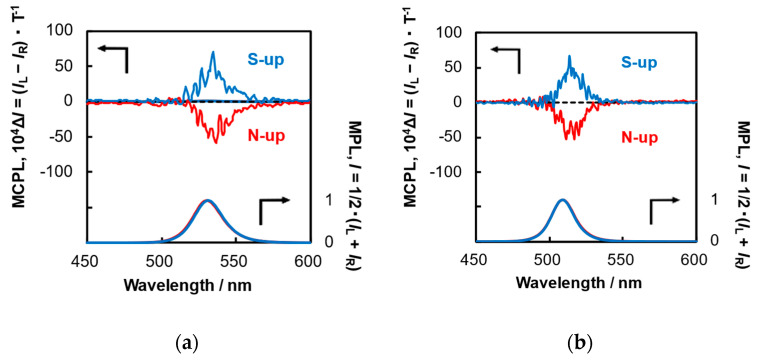
MCPL (upper spectrum in each panel) and MPL (lower spectrum in each panel) spectra of (**a**) PVQD-1 (λ_ex_ = 398 nm) and (**b**) PVQD-2 (λ_ex_ = 360 nm) in dilute toluene (1.0 × 10^−3^ M) under N-up (red lines) and S-up (blue lines) Faraday configurations within a magnetic field of 198 mT at 25 °C.

To investigate the relationship between the magnetic field strength and the field-induced chiroptical properties, an external magnetic field of 198 mT was applied to PVQD-1 and PVQD-2 using electromagnets, and their MCPL spectra were measured (see Figure 3 and Table 2).

As expected, when the magnetic field strength was increased to 158 mT, 198 mT, and 1.7 T [20], the MCPL wavelength shifted toward longer wavelengths and the magnitude of the anisotropy factor increased significantly. This result indicates that one of the factors contributing to MCPL is the mitigation of orbital degeneracy due to Zeeman splitting [27].

**Figure 4:**
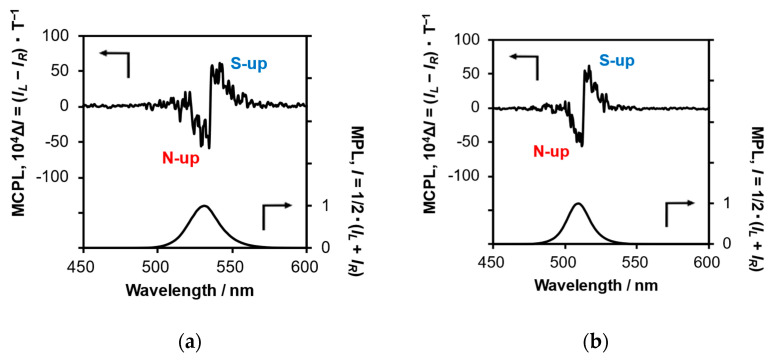
Switching MCPL (upper spectrum in each panel) and MPL (lower spectrum in each panel) spectra of (**a**) PVQD-1 (λ_ex_ = 398 nm) and (**b**) PVQD-2 (λ_ex_ = 360 nm) in dilute toluene (1.0 × 10^−3^ M) in a 198-mT Faraday configuration magnetic field at 25 °C. The switching wavelength is 537 and 513 nm for PVQD-1 and PVQD-2, respectively.

Next, to develop MCPL switching properties in the PVQDs, the direction of the magnetic field was abruptly reversed from S-up to N-up at the peak MCPL wavelength for PVQD-1 and PVQD-2 in a 198-mT external magnetic field while scanning from the long- to short-wavelength regions during MCPL measurements (Figure 4).

Consequently, the sign of the MCPL spectra of PVQD-1 and PVQD-2 changed from positive (+) to negative (−) at the maximum wavelength of the MCPL. These results include the magnetic hysteresis phenomena of PVQD-1 and PVQD-2, but they suggest that the rotation direction of MCPL can be instantaneously switched by changing the direction of the applied magnetic field.

**Figure 5:**
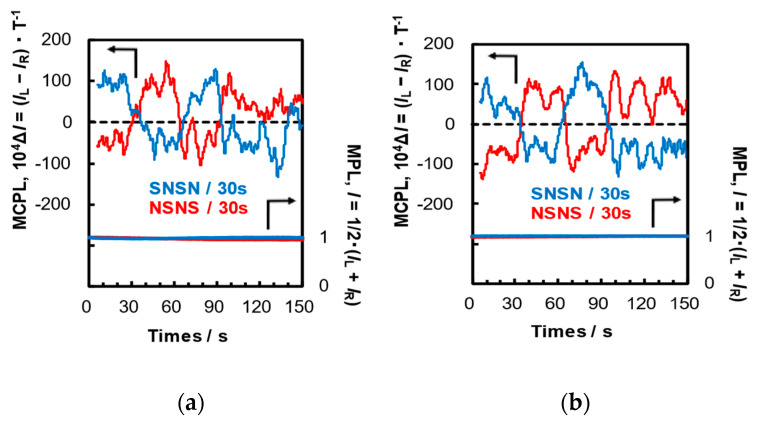
Reversible-switching MCPL (upper spectrum in each panel) and MPL (lower spectrum in each panel) plots of (**a**) PVQD-1 at 534 nm (λ_ex_ = 398 nm) and (**b**) PVQD-2 at 515 nm (λ_ex_ = 360 nm) in dilute toluene (1.0 × 10^−3^ M) in a 158-mT Faraday configuration magnetic field at 25 °C. The magnetic field is reversibly switched every 30 s.

Next, we attempted continuous and reversible switching of the rotation direction of MCPL by continuously changing the direction of the external magnetic field applied to the PVQDs. For PVQD-1 and PVQD-2, we applied an external magnetic field of 158 mT while reversibly switching its direction every 30 s, and plotted the MCPL intensity at 534 nm for PVQD-1 and 515 nm for PVQD-2. The measurement results are shown in Figure 5, and the reversible switching MCPL characteristics are listed in Table 3. In the MCPL measurements, we started scanning from the long wavelength side in the S-up magnetic configuration, while changing the direction of the applied magnetic field three times (N-up, S-up, N-up). As expected, the sign of the MCPL plots shifted sequentially from positive (+) to negative (−) and back to positive (+) and again to negative (−) as the magnetic field transitioned from S-up to N-up and then reversed.

Similarly, the direction of the magnetic field applied to PVQD-1 and PVQD-2 was switched three times in succession (S-up, N-up, and S-up) starting from the N-up configuration, and the MCPL plots were measured. As expected, an instantaneous switch between the N-up and S-up configurations using electromagnets changed the sign of the MCPL plots from negative (−) to positive (+) and then again from negative (−) to positive (+), resulting in a spectrum with mirror symmetry.

These results indicate that the direction of rotation of MCPL from PVQD-1 and PVQD-2 can be switched reversibly and instantaneously by reversibly switching the direction of the applied magnetic field.

We propose the following explanation for the effect of magnetic field direction on the rotation direction of MCPL. The luminescent mechanism of PVQDs PVQD-1 and PVQD-2 is the recombination of Pb^II^ excitons even under external magnetic field conditions. The difference between CH_5_N_2_CH_5_N_2_^+^ and CsCs^+^ changed the band gap of the orbital of the PVQDs, thereby mitigating orbital degeneracy, which may have changed the wavelengths of the MCPL and MPL spectra. Furthermore, the instantaneous switching of the direction of the applied magnetic field may have changed the spin state of the excited electrons and reversed the rotational direction of the MCPL.

## 3. Materials and Methods

### 3.1. Materials

PVQD-1 (Lot No. MKCM8860), consisting of CH_5_N_2_PbBr_3_, and PVQD-2 (Lot No. MKCN9570), consisting of CsPbBr_3_, were sourced from Sigma-Aldrich Japan (Tokyo, Japan). PVQD-1 and PVQD-2 were treated with oleic acid and oleylamine. The average core size for both samples was approximately 10 nm.

### 3.2. MPL and MCPL Spectroscopy

MCPL and MPL spectra were measured using a JASCO (Hachioji, Tokyo, Japan) CPL-300 spectrofluoropolarimeter, which was equipped with electromagnets of 158 and 198 mT, at a scattering angle of 0°. A magnetic field was applied using a TESLA (Tokyo, Japan) TSMP182-1004015-1 electromagnet. This electromagnet is composed of a Helmholtz coil and is fixed to the JASCO CPL-300 in the Faraday configuration. The direction of the magnetic field can be controlled by changing the direction of the current flowing through the coil. Excitation was performed with unpolarized monochromatic light (bandwidth = 10 nm) at 25 °C. To quantitatively assess the magnitude of the MCPL efficiency (*g_MCPL_*), we utilized Equation (1).

(1) $$g_{MCPL}=\frac{\left(I_{L}-I_{R}\right)}{\left(I_{L}+I_{R}\right)/2}$$

*I_L_* and *I_R_* in Equation (1) represent the intensities of left- and right-handed MCPL, respectively, when excited by unpolarized light. The excitation wavelengths for the toluene solution (with a path length of 5 mm) were 398 nm for PVQD-1 and 360 nm for PVQD-2.

## 4. Conclusions

In this study, we demonstrated that two achiral antimagnetic perovskite-type PbQDs (CH_5_N_2_PbBr_3_ and CsPbBr_3_) produced bipolar MCPL spectra on the order of |*g*_MCPL_| = 10^−3^ in a dilute toluene solution at 25 °C when the N- and S-up directions of the Faraday configuration were applied in 158 and 198 mT magnetic fields. The value of the anisotropy factor increased with the magnetic field. Furthermore, we verified that the sign of the MCP spectrum could be controlled using the N- and S-up directions of the Faraday configuration and that the sign of the MCP spectrum could be reversibly switched by instantaneously switching the N and S poles. Based on this finding, we believe that MCP-PeLEDs that can freely switch between right- and left-rotating light can be developed by incorporating an appropriate MCP-perovskite light luminophore into a commercially available LED. In the future, we intend to demonstrate the feasibility of MCP-PeLEDs based on perovskite-type PbQDs.

## Figures and Tables

**Table 1:** MCPL properties for PVQD-1 and PVQD-2 in dilute toluene (1.0 × 10^−3^ M) under 158 mT at 25 °C.

PVQDs	Faraday Configuration	*λ*_ex_ (nm)	*λ*_MCPL_ (nm)	*g*_MCPL_ /10^−3^ (T^−1^)
1	N-up	398	535	−9.2
	S-up		537	+9.3
2	N-up	360	512	−5.0
	S-up		516	+7.9

**Table 2:** Magnetic field dependence of MCPL properties for PVQD-1 and PVQD-2 in dilute toluene (1.0 × 10^−3^ M) at 25 °C.

PVQDs	Magnetic Field Strength (T)	*λ*_MCPL_ (nm)	|*g*_MCPL_| /10^−3^
1	1.7 [20]	538	9.43
	0.198	537	1.53
	0.158	536	1.46
2	1.7 [20]	514	9.37
	0.198	513	1.13
	0.158	514	1.02

**Table 3:** Reversible-switching MCPL properties of PVQD-1 and PVQD-2 in dilute toluene (1.0 × 10^−3^ M) at 25 °C.

PVQDs		Faraday Configuration	15 min	45 min	75 min	105 min
1	Δ*I*	SNSN	+83	−35	+62	−39
	*g*_MCPL_ /10^−3^ (T^−1^)		+8.4	−3.6	+6.3	−3.9
	Δ*I*	NSNS	−39	+83	−39	+45
	*g*_MCPL_ /10^−3^ (T^−1^)		−3.9	+8.5	−4.1	+4.7
2	Δ*I*	SNSN	+51	−56	+93	−66
	*g*_MCPL_ /10^−3^ (T^−1^)		+5.1	−5.6	+9.4	−6.6
	Δ*I*	NSNS	−81	+72	−62	+71
	*g*_MCPL_ /10^−3^ (T^−1^)		−8.3	+7.3	−6.3	+7.1

## Data Availability

Data are contained within the article.

## Abbreviations

The following abbreviations are used in this manuscript:

CPL	Circularly polarized luminescence
MCPL	Magnetic circularly polarized luminescence
MPL	Magnetic photoluminescence
PVQD	Perovskite quantum dot
